# Machine Learning in Contaminated Soil Remediation: From Monitoring, Source Apportionment, and Risk Assessment to Practical Applications

**DOI:** 10.3390/toxics14100854

**Published:** 2026-09-25

**Authors:** Chen Li, Hong Jiang, Qi Deng, Linlin Li, Yankai Huang, Boyue Liu, Cuilian Fu, Jie Li

**Affiliations:** 1 Department of Basic Education, Shangqiu Institute of Technology, Shangqiu 476000, China; 2 School of Environmental and Municipal Engineering, Tianjin Chengjian University, Tianjin 300384, China; 3 State Key Laboratory of Biobased Fiber Manufacturing Technology, Tianjin University of Science and Technology, Tianjin 300457, China

**Keywords:** historical data analysis, soil monitoring data, scenario simulation, data exploitation

## Abstract

Severe soil contamination over recent years has sparked massive research focus on soil remediation. Traditional remediation approaches are categorized as physical, chemical, and biological methods, alongside their composite combined remediation modes. Matching remediation strategies to site-specific pollution features facilitates enhanced remediation efficiency. Nonetheless, complicated soil matrices and heterogeneous pollutants in terms of types, physicochemical traits, and concentrations render the screening and optimization of remediation technologies highly challenging. Recent advances have facilitated the widespread application of artificial intelligence (AI) in soil remediation research. Machine learning (ML), as a core branch of AI, substantially enhances the management and analytical performance of soil monitoring data. By collating and analyzing literature related to soil remediation, machine learning, and artificial intelligence, this review outlines state-of-the-art advances in ML-driven data processing and remediation optimization and further compares the performance and characteristic properties of various machine learning models for soil remediation across different pollutant categories. Machine learning leverages historical data analysis, scenario simulation and nature-inspired optimization strategies to greatly reduce soil sampling requirements and improve data utilization efficiency. This review summarizes the key strengths and inherent drawbacks of machine learning techniques in contaminated soil remediation and further indicates the promising application prospects of machine learning in this field.

## 1. Introduction

Recent decades have witnessed rapid industrial advancement and intensive agricultural activities, exerting pronounced anthropogenic impacts on soil ecosystems. Soil contamination rarely originates from a single isolated driving factor; rather, it stems from the coupled interactions of diverse chemical, physical, and biological processes [1]. Soil contaminants are generally categorized into two primary groups: organic contaminants and inorganic contaminants. Typical representatives of organic contaminants include polycyclic aromatic hydrocarbons, petroleum hydrocarbons and a range of pesticides. Owing to their slow degradation kinetics in environmental matrices, such substances can persist within soil compartments over extended timescales [2,3]. Characterized by miniature particle sizes and high mobility, microplastics have drawn growing scholarly focus in recent years, as they are able to spread within soil systems and induce relevant ecological impacts [4]. Toxic inorganic contaminants include heavy metals such as cadmium and lead, alongside metalloid arsenic, all of which are prone to long-term persistence and gradual accumulation in soil systems [5,6,7]; furthermore, some inorganic non-metallic contaminants, such as fluoride and selenium, show strong bioavailability in particular environmental scenarios [8,9]. Taken as a whole, these multiple factors substantially increase the complexity of soil contamination. Given the growing complexity of soil contamination scenarios, targeted and effective management of soil contaminants is urgently required. Accordingly, a wide range of remediation approaches have been developed worldwide to address this challenge. Currently, physical, chemical and biological remediation represent the three most widely adopted treatment strategies. As a typical example of physical remediation, thermal desorption can remove part of soil contaminants through thermal treatment. Nevertheless, this technique usually requires high energy input and may impose adverse impacts on soil architecture during treatment [10]. Chemical remediation approaches represented by redox reactions have been extensively deployed at numerous contaminated sites. Nevertheless, intermediate by-products may form in the course of remediation, and such substances could trigger latent environmental hazards [11]. Bioremediation predominantly utilizes microorganisms and vegetation to degrade and immobilize soil contaminants, which accordingly renders it an environmentally benign remediation strategy. Nevertheless, its remediation efficiency is vulnerable to a series of variables such as temperature, soil moisture, and nutrient supply. Accordingly, the whole remediation cycle proceeds at a low rate and presents unstable performance [12]. Conventional remediation technologies typically rely on iterative trial-and-error testing to determine optimal operational parameters for field deployment. When soils harbor multiple contaminants and exhibit strong spatial heterogeneity, these approaches often face difficulties balancing treatment efficiency and remediation precision [13]. Given the complex nature of prevailing soil contamination scenarios, the development of targeted remediation strategies and the improvement of treatment efficiency stand as core research priorities for forthcoming soil environmental investigations.

Confronted with increasingly complex challenges in the field of soil remediation, machine learning (ML) has attracted growing scholarly interest owing to its robust data manipulation capabilities. Specifically, supervised learning is widely employed for regression and classification tasks, while unsupervised learning serves as a standard tool for dimensionality reduction and cluster analysis [14]. By contrast, reinforcement learning is mainly deployed to address decision-making issues demanding continuous feedback and adaptive tuning [15]. Machine learning (ML) has been introduced into soil contamination research to interpret complex nonlinear correlations between environmental factors and contaminant dynamics, covering spatial contamination forecasting, source apportionment, and efficient mining of multi-source datasets [16]. Moreover, ML enables the comprehensive evaluation of contamination’s potential risks to eco-environments and public health by coupling diverse data sources [17,18].

In recent years, a growing body of literature has investigated the application of machine learning approaches in this field, using data analytics to address the limitations of conventional research techniques when characterizing complex environmental conditions. Early studies generally relied on empirical inference or standalone models to interpret contaminant migration behaviors. Nevertheless, soil systems in real field scenarios exhibit prominent multi-factor coupling characteristics, and such approaches fail to fully capture their variation patterns [19,20]. By contrast, machine learning places greater emphasis on mining implicit information from datasets and uncovering latent correlations among variables via training on massive sample datasets. In practical field scenarios, machine learning is capable of integrating multi-source data including soil physicochemical properties, contaminant traits, environmental parameters, and historical remediation archives, so as to further explore the latent nonlinear correlations hidden within these datasets [21]. With such data-driven approaches, researchers can not only characterize contamination status with higher precision but also conduct simulation analyses of remediation processes. To some extent, these analytical outputs provide reliable references for the selection of optimal remediation strategies. Accordingly, machine learning has been increasingly applied across multiple research phases covering contamination monitoring, remediation process optimization and decision support. Consequently, relevant studies no longer rely entirely on conventional empirical trials but are gradually shifting toward data-driven and intelligent analytical paradigms [12,13]. Moreover, machine learning supports the fusion of multi-source datasets to advance intelligent decision-making. For instance, by synthesizing contaminant traits and site environmental conditions, as well as the treatment efficiency and economic expenditure of diverse remediation approaches, researchers can evaluate and screen optimal remediation strategies and construction schedules tailored to distinct contamination scenarios [22]. This strategy can not only improve overall remediation efficiency but also enable preliminary simulation of economic costs and environmental repercussions for different remediation tactics. Such predictive analyses support the selection of balanced solutions with optimal cost effectiveness, ensuring environmental security and substantially lowering time and capital inputs [23].

This review collates and synthesizes published literature on the application of machine learning techniques across all phases of contaminated site remediation. Organized by contaminant category, it summarizes research progress in machine learning for remediation of diverse contaminated soils, elaborates on respective advantages and limitations, and puts forward targeted research recommendations. This work provides systematic decision support for researchers selecting appropriate remediation strategies, to facilitate precise optimization and matching of remediation schemes under multi-objective constraints.

## 2. Overview of Machine Learning Methods

Machine learning constitutes a vital branch of artificial intelligence, and core research focuses on enabling computational models to capture implicit patterns from datasets and iteratively boost predictive performance based on such extracted patterns. With the continuous expansion of data volume and the advancement of computing capacity, machine learning has generated diverse learning algorithms and model architectures, thereby establishing a relatively complete methodological framework [24]. According to the characteristics of input data and learning paradigms, machine learning is generally classified into five major categories, namely, supervised learning, unsupervised learning, semi-supervised learning, reinforcement learning, and artificial neural networks (Figure 1).

Supervised learning represents a classic modeling paradigm within machine learning, wherein predictive models are trained using paired input data and corresponding labeled samples. During the training process, the algorithm iteratively calibrates its predicted outputs against the labeled values to minimize discrepancies between predictions and measured observations, in turn learning the underlying mapping relationships between input features and target outputs [25]. Statistical learning theory lays the theoretical foundation for supervised learning, whose core challenge lies in maintaining stable and accurate predictive performance of models on unseen datasets under the limitation of insufficient sample sizes [26]. In line with the attributes of output variables, supervised learning tasks are divided into two main categories: regression tasks for predicting continuous numerical values and classification tasks that yield results from a limited set of discrete categories [27]. To strengthen models’ capacity to fit complex nonlinear correlations, researchers have developed a variety of nonlinear supervised learning methods, including support vector machines, instance learning algorithms, and ensemble models built upon decision trees [28]. Unsupervised learning uses analytical models to explore the intrinsic structure and distribution properties of datasets independent of label information; different from supervised learning, its core task is not the direct prediction of target variables, but the excavation of latent data patterns through analyzing inter-sample similarities and statistical characteristics [29]. Cluster analysis stands as the most prevalently adopted unsupervised learning approach. It partitions datasets into multiple subgroups with high intra-group similarity by quantifying the similarity metrics between individual samples [30]. Semi-supervised learning constructs predictive models by leveraging a small set of labeled samples alongside large unlabeled datasets. It relies on two foundational assumptions: samples with comparable features fall into the same class, and data form distinct clusters across the feature space. Where these assumptions are valid, unlabeled data sharpens model decision boundaries and enhances overall predictive capability [31]. Reinforcement learning investigates how intelligent agents acquire optimal decision-making policies via continuous interactions within dynamic environments. Distinct from algorithms trained on static datasets, reinforcement learning highlights the influence of action choices on subsequent environmental states and reward signals, with its core objective formulated to maximize long-term cumulative rewards [32]. Reinforcement learning is built on four core elements across its learning loop: state, action, reward, and policy. An agent selects actions according to its current state and iteratively refines its policy using environmental feedback. Over recent decades, the integration of deep learning into reinforcement learning architectures has substantially enhanced their adaptability to high-dimensional state spaces and complex decision scenarios [33]. Artificial neural networks are a class of nonlinear modeling approaches inspired by biological neural systems, composed of large numbers of interconnected neuron units. Rather than forming an independent learning category, they function as a flexible model architecture applicable to supervised, unsupervised, semi-supervised, and reinforcement learning paradigms. Each neuron computes a weighted sum of input signals and performs nonlinear transformation through activation functions. This structural property enables such networks to accurately approximate complex functional relationships between variables [34]. Neural networks typically adopt a three-layer architecture including input, hidden, and output tiers. Raw features are captured by the input layer, stepwise feature transformation and integration are executed within hidden layers, and conclusive outputs are derived from the output layer. With the continuous advancement of relevant research, neural networks have evolved from shallow architectures into deep models stacked with multiple hidden layers, a research field commonly referred to as deep learning (DL) [35]. Through layer-by-layer mapping, deep learning hierarchically distills features and allows models to self-learn high-level semantic abstractions from raw low-level information. Versus shallow architectures, it delivers outstanding performance in feature extraction and expressive power [36]. In general, artificial neural networks and deep learning architectures serve as versatile modeling tools capable of autonomous feature extraction and predictive modeling across multiple machine learning paradigms. Carefully designed network architectures, training schemes and regularization strategies allow these models to exhibit high flexibility in handling high-dimensional data and complex pattern recognition tasks [37].

## 3. Machine Learning in Soil Remediation

As a core branch of artificial intelligence, machine learning leverages data-driven models to describe contaminant transport and transformation, optimize remediation operating conditions, and predict treatment technology performance. This makes it a highly promising tool for environmental engineering applications. Widely used algorithms such as random forest (RF), artificial neural networks (ANNs), and long short-term memory (LSTM) networks now support a broad range of soil remediation applications, including site characterization, pollution risk assessment, technology selection, and process control [38], which substantially enhances the prediction accuracy and economic efficiency of soil remediation projects. In general, the application scope of machine learning in soil remediation keeps expanding, which covers not only pollution remediation and spatial distribution prediction, but also remediation strategy optimization, treatment efficiency assessment, pollution risk identification, and other relevant research directions. The bibliometric analysis for annual publication trends (Figure 2) was conducted on the Web of Science Core Collection. The Boolean retrieval query was TS = (“soil remediation”). Only English articles and reviews were included. The retrieval cutoff date was 30 April 2026. After duplicate removal, a total of 32,874 soil-remediation-related bibliographic records spanning 2012–2026 were obtained. Through an annual analysis of 32,874 articles from the Web of Science database between 2012 and 2026, this provides a clear illustration of the annual publication volume and growth rate in soil remediation and machine learning research, as shown in Figure 2.

Publications focusing on machine learning applications for soil remediation exhibited a sustained increasing trend from 2012 to 2025. Broadly, publications applying machine learning to soil remediation research climbed from a single paper in 2012 to 98 outputs in 2025, revealing progressively growing research attention devoted to this topic. During this period, publication growth remained nearly stagnant from 2012 to 2020, which implies that machine learning was still in the preliminary exploratory stage with regard to soil remediation research. Nevertheless, the speed of growth started to pick up in 2021, as artificial intelligence gradually overcame early developmental bottlenecks, allowing continuous optimization of machine learning algorithms and the creation of models customized for soil remediation scenarios. Over this timeframe, the total quantity of papers focused on soil remediation grew steadily and reached 3594 by 2025, presenting a general upward tendency. Although a minor drop in soil remediation publications occurred in 2023, the overall output of this field still trended upwards. Meanwhile, the growth rate of studies integrating machine learning and soil remediation has kept rising in recent years. Accordingly, optimizing and upgrading soil remediation processes via machine learning techniques remains a central research priority in this discipline. Note that the data for 2026 only included publications collected by 30 April 2026 and this does not represent a full-year statistic.

An independent literature retrieval was performed in the Web of Science Core Collection for keyword visualization (Figure 3). The Boolean search query was TS = (“soil remediation”) AND TS = (“machine learning” OR “artificial intelligence”). Only English articles and reviews published from 2017 to 2025 were retained. This separate search obtained 758 relevant publications. With the aid of VOSviewer software (version 1.6.20), full counting was adopted as the counting method. High-frequency keywords with a minimum occurrence threshold of 12 were extracted from the titles, abstracts, and author-supplied keywords of the collected publications. The association strength normalization method was used, and the default cluster settings of VOSviewer were applied for visual analysis to intuitively characterize the research hotspots of this field, as displayed in Figure 3.

Machine learning effectively addresses the limitations of traditional analytical and remediation approaches, such as low operational efficiency and high vulnerability to environmental disturbances. Random forest (RF), artificial neural networks (ANNs), Extreme Gradient Boosting (XGBoost), and support vector machines (SVMs) are the most widely used algorithms in related modeling studies. Built on multi-source data fusion, existing studies mainly focus on soil heavy metal pollution monitoring, spatial distribution prediction, model interpretability improvement, and remediation scheme optimization. The applications of machine learning within soil remediation concentrate on three core research categories: heavy metal immobilization, degradation of organic contaminants, and plant–microbe synergistic remediation. Current research scenarios include mining-disturbed agricultural lands, regions affected by petroleum exploitation, storage and transportation, and industrial contaminated sites. Machine learning enables soil pollution source identification, remediation technology optimization, and ecological risk assessment via the development of high-precision predictive models. Building on the capabilities described above, machine learning can further forecast the adsorption characteristics and elucidate the intrinsic remediation mechanisms of novel functional materials, particularly, biochar and nanomaterials. In addition to these established applications, machine learning is increasingly driving advances in three emerging directions: spatial simulation modeling, intelligent regulation of remediation strategies, and uncertainty analysis. Collectively, these technical advances have been shown to improve remediation efficiency while reducing associated costs, thereby promoting the intelligent and precise transformation of modern soil remediation technologies. This review adopts a contaminant classification framework to review the current status of machine learning applications in various types of soil remediation, and it is intended to serve as a comprehensive reference for research and engineering practice in this field.

To identify representative studies for detailed analysis and tabulation, we conducted a stepwise screening based on the 758 publications retrieved from the Web of Science Core Collection. First, title and abstract screening was performed to exclude studies that did not address soil remediation applications, such as pure methodological development of machine learning algorithms, soil quality monitoring without remediation design, and review articles. This step retained original research articles focusing on machine learning applications across the full workflow of soil remediation. Second, full text assessment was carried out with predefined inclusion criteria. Studies were included if they reported specific model architectures, quantitative performance metrics, and clearly defined soil contaminant types and remediation techniques. Studies were excluded if they only presented conceptual frameworks, lacked reproducible performance data, or focused solely on pollution source apportionment and risk assessment without remediation relevance. Representative studies from each contaminant category were then selected for systematic collation and in-depth discussion. Specifically, seven studies on petroleum contaminant remediation and 16 studies on inorganic contaminant remediation have been organized into Table 1 and Table 2, respectively, to present comparative performance information.

### 3.1. Machine Learning in Organic Contaminant Remediation

Against a backdrop of diverse soil organic contaminants with distinct contamination characteristics, intelligent technologies offer innovative solutions to overcome the inherent drawbacks of conventional remediation approaches. Deep learning architectures represented by three dimensional convolutional neural networks (3DCNNs) are capable of predicting contaminant concentrations and spatial distribution patterns by incorporating soil physicochemical properties, which can serve as a methodological reference for investigations into organic contamination [39]. Machine learning has been validated as an efficient technique for assessing the performance of chemical oxidation remediation and quantifying dominant influencing factors [40]. Moreover, the integration of machine learning and numerical analysis facilitates multi-objective optimization of field-scale remediation strategies [41]. This section focuses on four major categories of organic contaminants: petroleum-derived pollutants, pesticide residues, industrial solvents, and microplastics. For each contaminant category, it details specific machine learning application frameworks for contaminant distribution prediction and treatment performance forecasting. By synthesizing existing research cases, we compare the predictive performance of diverse algorithms, summarize key advances and prevailing bottlenecks of relevant applications, and outline future development trends of machine learning for targeted remediation of organically contaminated soils.

#### 3.1.1. Machine Learning in Petroleum Contaminant Remediation

Petroleum-derived contaminants released into soil during extraction, transportation, and other industrial activities are among the most prevalent and harmful organic contaminants in subsurface environments. Their key constituents include total petroleum hydrocarbons (TPH); benzene, toluene, ethylbenzene, and xylenes (BTEX); and polycyclic aromatic hydrocarbons (PAHs). These contaminants are typically characterized by high hydrophobicity, low environmental degradability and strong biotoxicity. In field conditions, they are distributed heterogeneously. Coupled with the inherent heterogeneity of soil matrices and the synergistic effects of diverse environmental factors, this makes the remediation process highly complex and unpredictable. Representative research cases on machine learning applications for petroleum-contaminated soil are summarized in Table 1 below.

**Table 1:** Major application scenarios of machine learning in petroleum contaminant remediation.

Contaminant(s) Under Study	Sample Size	Model(s) Used	Model Input Parameters	Model Accuracy Parameters	Objective	Result	Reference
PAHs, TPH	545 data points; randomly split into 80% training set and 20% test set	TPOT, GBM, DRF, GLM, DNN	Remediation technology, soil moisture, incubation period, type and concentration of petroleum hydrocarbon (PH), soil organic carbon, organic nitrogen, phosphate	MSE, RMSE	AutoML (8 variables) compares TPOT and H_2_O on petroleum-contaminated soil bioremediation data	H_2_O AutoML (GBM) has lower MSE (11.36%^2^) than TPOT (16.39%^2^)	[12]
PAHs	1422 experimental records; stratified into 80% training set and 20% test set	XGBoost, RF, ANN, PDP, SHAP	Pollutant properties (octanol–water partition coefficient Kow, solubility, molecular weight, initial concentration), remediation reagents (oxidant type and concentration, catalyst, inhibitor, eluent), soil properties (moisture, pH, water–soil ratio), remediation duration	R^2^, RMSE, PCC	Build interpretable models for PAH removal prediction	Ferrous-activated persulfate removes 93.4% of benzo[a]pyrene.	[40]
PAHs	217 experimental data points	RF, SVR, BPNN, XGB	Microwave parameters (treatment time, power, temperature), soil properties (density, pH, moisture, organic matter, specific surface area, oil-soil mass ratio), initial total petroleum hydrocarbon (TPH) concentration	R^2^, RMSE	SHAP and partial dependence analysis guide parameter optimization	Optimized TPE model (R^2^ = 0.9658, RMSE = 4.58%) outperforms RF, SVR and BPNN	[42]
PAHs, PHCs, BaP	914 data points; stratified random split into 80% training set and 20% test set	SVM, XGB, RF, ANN	Soil physicochemical properties (pH, base saturation, cation exchange capacity (CEC), effective CEC, C/N ratio, total nitrogen, sand/silt/clay fractions, soil humidity), climatic conditions, spatiotemporal attributes, ecological function indicators	R^2^, NRMSE, SD, PFI	ML analyzes soil properties, NPP and drivers of greenhouse gas emissions	The random forest model performed best (R^2^ = 0.754, NRMSE = 0.098).	[43]
PHCs	25 independent laboratory experiments; 10,000 random parameter samples generated by Monte Carlo simulation	RANSAC, MCO, RF, KNN, LR	Solution pH, soil moisture, applied voltage, sodium persulfate concentration, surfactant dosage, ultrasonic treatment time	R^2^, MSE, RMSE, MAE, SD, KDE, IQR	AI optimizes coupled process for petroleum hydrocarbons removal	RANSAC shows strong generalization (R^2^ = 0.90 for train & test)	[44]
PAHs, BTEX	2394 data points extracted from 48 peer-reviewed experimental studies	CB, RF, DT, XGB, SVR, ANN	Surfactant properties (concentration, hydrophilic–lipophilic balance (HLB), molecular weight, critical micelle concentration (CMC), interfacial tension), soil properties (particle composition, organic carbon, organic matter, pH, cation exchange capacity (CEC), permeability, bulk density, porosity), oil pollution parameters (concentration, viscosity, density, asphaltene content), water chemistry (pH, hardness), operation conditions (stirring speed, washing time, temperature, liquid–soil ratio)	RMSE, MAE, AARE, NRMSE, SD, R, R^2^	Six ML models for oil removal prediction	96.41% predictions within acceptable range	[21]
PAHs	5803 batch experimental data; randomly split into 80% training set and 20% test set; supplemented with field-contaminated soil samples from Hangzhou for validation	XGB, RF, SVM, ANN	Soil properties (bulk density, moisture, organic matter content), thermal desorption process parameters, polycyclic aromatic hydrocarbon (PAH) pollutant characteristics	R^2^, RMSE	PAH removal model validated, key factors analyzed via SHAP	XGBoost performs best; key factors: curing time, temperature, C0, moisture content	[45]

(Note: TPOT = Tree-Based Pipeline Optimization Tool; GBM = Gradient Boosting Machine; DRF = distributed random forest; GLM = generalized linear model; DNN = deep neural network; MSE = mean squared error; RMSE = root mean squared error; PDP = partial dependence plot; SHAP = Shapley additive explanations; PCC = Pearson correlation coefficient; SVR = support vector regression; BPNN = back propagation neural network; XGB = Extreme Gradient Boosting; NRMSE = normalized root mean squared error; SD = standard deviation; PFI = permutation feature importance; RANSAC = random sample consensus; MCO = Monte Carlo optimization; KNN = K-nearest neighbors; LR = logistic regression; MAE = mean absolute error; KDE = kernel density estimation; IQR = interquartile range; CB = CatBoost; DT = decision tree; AARE = average absolute relative error; R = correlation coefficient; R^2^ = coefficient of determination).

Cross study comparison of the cases in Table 1 reveals several consistent patterns. Ensemble learning algorithms, particularly XGBoost, random forest, and Gradient Boosting Machine, consistently deliver the highest predictive accuracy across most datasets, with R^2^ values generally above 0.85 and lower prediction error relative to single models such as support vector machines and back propagation neural networks. In terms of contaminant coverage, research concentrates heavily on PAHs and total petroleum hydrocarbons, while investigations targeting BTEX and other individual petroleum fractions remain relatively scarce. Soil physicochemical properties, remediation operational parameters and initial contaminant concentration are identified as the most widely adopted input features across all models. Most studies are based on laboratory batch experimental data, and field validated models for actual site remediation remain limited.

Within the scope of thermal remediation technologies, microwave heating has garnered growing research interest owing to its outstanding treatment efficiency and relatively low energy input. Relevant experimental results reveal that the Bayesian-optimized XGBoost model achieved the optimal predictive performance with a coefficient of determination of 0.9658. That study also confirmed that microwave heating temperature exerted the dominant influence on remediation performance [42]. Nevertheless, the model developed in that work suffers from several drawbacks, such as insufficient representativeness of datasets, single evaluation metric and incomplete coverage of characteristic variables. Its generalization capacity and engineering practicability remain to be further enhanced. Moreover, Chen et al. [10] employed machine learning algorithms including random forest to simulate thermal desorption remediation processes for PAH-contaminated soils. Their model is capable of not only forecasting remediation efficiency but also quantifying the contributions of various parameters such as heating regimes and contaminant intrinsic properties. Persulfate-based advanced oxidation processes are extensively adopted within the framework of chemical oxidation remediation. Recent investigations have leveraged machine learning to optimize integrated remediation techniques, particularly for the simultaneous treatment of petroleum-contaminated soils via coupled electrolysis, ultrasonic activation, and persulfate oxidation. By integrating random sample consensus regression with Monte Carlo optimization, researchers conducted fine tuning on six core parameters and determined optimal operational conditions capable of achieving a contaminant removal efficiency ranging from 70% to 90% [44]. The treatment performance of surfactant-enhanced remediation is controlled by a range of factors, including soil texture, inherent contaminant properties, surfactant composition, and operational parameters. A study compiling 2394 experimental datasets from 48 published works showed that machine learning models based on categorical boosting algorithms can reliably predict oil removal efficiency. It also identified several dominant influencing factors, including stirring speed, surfactant dosage, and liquid-to-soil ratio [21].

Across thermal remediation, chemical oxidation, and surfactant flushing alike, machine learning supports the regulation of process parameters and the identification of dominant influencing variables. It enables the processing of complex datasets and the extraction of implicit internal patterns. Expanding input datasets helps build more intelligent remediation decision-making frameworks. Machine learning advances the remediation of petroleum-contaminated soils across three core dimensions: contaminant monitoring and diagnosis, remediation process optimization, and remediation strategy development. By quantifying and forecasting the traditionally empirical black-box nature of operational processes, these approaches enhance remediation process controllability. Accordingly, they deliver efficient and cost-effective solutions to growing petroleum contamination challenges, and they outperform conventional empirical remediation strategies.

#### 3.1.2. Machine Learning in Pesticide Contaminant Remediation

Organophosphate and organochlorine pesticides are extensively applied in agricultural practices and exert prominent effects on pest and disease management. Nevertheless, such agrochemicals feature high biotoxicity, low environmental degradability and strong bioaccumulation potential. Once introduced into soil matrices, these agrochemicals induce persistent residual risks and pose latent hazards to ecological balance and food safety [46]. At present, the primary remediation technologies for this type of contamination include chemical oxidation and microbial degradation. However, these approaches still face several limitations in laboratory research and field-scale application. For example, they typically involve long remediation cycles and pose secondary contamination risks throughout the treatment process. Furthermore, the coexistence of multiple pesticide residues in soil matrices further adds to the complexity of the entire remediation workflow.

Within the realm of soil contamination remediation, gradient boosting regression trees (GBRT) and support vector regression (SVR) have been extensively utilized to establish quantitative correlations between pesticide degradation behaviors and core influencing variables including soil physicochemical characteristics and climatic conditions based on abundant experimental datasets. Accordingly, such algorithms are competent to forecast the degradation half-lives as well as migration and transformation behaviors of pesticides. For instance, Shen et al. [47] constructed a GBRT-based machine learning model with a dataset consisting of 1363 samples to predict the degradation half-lives of pesticides within plant systems. Experimental outcomes revealed that the optimal GBRT-ECFP model yielded a classification score of 0.698 ± 0.010, surpassing alternative algorithms. This model enables efficient differentiation of pesticides with distinct half-lives, providing a solid basis for pesticide environmental risk assessment and agricultural management. Moreover, ensemble learning algorithms such as random forest and support vector regression (SVR) have been extensively applied to explore pesticide degradation pathways. Through multivariate data analysis, these methods can identify the dominant variables governing degradation rates, including soil pH, organic matter content, ambient temperature, and soil water content [48].

In recent years, relevant research has explored the application of machine learning (ML) methods to soil pesticide remediation. These methods are particularly effective at analyzing complex data relationships and identifying patterns in existing data, thereby offering guidance for parameter selection and design during the remediation process. Such models can simulate dynamic changes in contaminant concentrations under various environmental conditions to support the development of soil remediation plans [10,49]; Furthermore, existing literature reviews indicate that incorporating relevant physical mechanisms into machine learning modeling processes can enhance the interpretability of such models and provide new directions for the development of precise soil remediation technologies [50]. In this context, relevant research no longer relies solely on empirical judgment but incorporates additional data [46,51].

#### 3.1.3. Machine Learning in Industrial Solvent Contaminant Remediation

Solvent-borne contaminants are widespread across industrial contaminated sites. They comprise primarily volatile organic compounds (VOCs), including trichloroethylene (TCE), perchloroethylene (PCE), and dichloromethane, alongside methanol and BTEX fractions such as benzene and toluene. These compounds exhibit high volatility, readily partitioning from contaminated soil and groundwater into the atmosphere and posing sustained threats to ambient ecosystems and human health. The transport, diffusion, and transformation of VOCs within soil matrices are inherently complex processes. As a result, conventional remediation approaches typically suffer from excessive energy consumption and elevated operating costs at field scale [52,53]; remediation efficiency drops further when co-occurring solvent contaminants are present or when soil hydraulic permeability is low. Additionally, degradation of certain solvents generates more toxic intermediate byproducts, which frequently give rise to secondary contamination [54,55].

Convolutional neural networks (CNNs) specialize in processing spatially structured data, making them widely adopted to map VOC concentration distribution patterns across soil sampling sites. Recurrent neural networks (RNNs) and their variants such as long short-term memory (LSTM) are optimized for time-series analysis and are particularly suitable for investigating dynamic contaminant migration processes in soil. Trained on available monitoring and experimental datasets, these models accurately capture VOC distribution and variation patterns in soil systems, and support predictions of treatment performance across a range of remediation technologies, including physical adsorption, chemical oxidation, and biodegradation [10]. In another study, Sulaiman et al. [56] applied an Elman neural network (ENN) to model and optimize the biofiltration process of mixed VOCs; the results indicate that this neural network is well-suited for complex reaction kinetics problems. Beyond the independent deployment of individual models, such machine learning algorithms can integrate multi-source datasets covering geology, hydrology, chemistry, and engineering disciplines, to facilitate the tracing and precise localization of latent contaminant sources.

In field-scale soil remediation practices, such models enable virtual simulation of a wide range of remediation schemes. Comparative analysis of simulated outputs facilitates the screening of the most feasible remediation schemes, reduces operational expenditure, and fulfills predefined environmental remediation targets.

#### 3.1.4. Machine Learning in Soil Microplastic Contamination Remediation

Microplastics are featured by tiny particle sizes, chemical stability, and strong environmental persistence. Accordingly, such contaminants pose grave latent hazards to soil ecological matrices [57]. They not only damage soil physical structure and disrupt nutrient cycling and water retention capacity but also act as adsorption carriers for other toxic substances. These harmful particles can migrate along the food chain, ultimately threatening ecological security and human health. However, microplastics in soil matrices have highly heterogeneous distribution and complex compositions. Coupled with the inherent limitations of conventional detection and remediation techniques such as physical sieving and chemical leaching, achieving efficient and accurate monitoring and management of these contaminants remains a major challenge in environmental research.

For contaminant monitoring and source apportionment, these algorithms handle large multi-source datasets from remote sensing satellites, in situ sensors, and hyperspectral imaging systems. Image recognition tools enable rapid preliminary screening of microplastics across extensive soil areas and help pinpoint latent contamination hotspots. Beyond monitoring, machine learning also drives advances in remediation material development and treatment technology optimization. For example, in the development of novel bioremediation materials, generative adversarial networks (GANs) are used to augment available plastic-degrading enzyme sequence data, while pre-trained language models mine metagenomic databases for high-potential PET-degrading enzymes. These strategies have markedly expedited the discovery of high-performance bioremediation agents [58]. Machine learning coupled with design of experiments (DoE) shows strong potential for use in physical and chemical remediation systems. One published study, for example, examined polystyrene microplastic removal from aqueous solutions using magnetic nanoadsorbents. The research team first generated preliminary datasets through experimental design, then applied classical algorithms including support vector machine (SVM), random forest (RF), and artificial neural network (ANN) to perform multi-objective optimization. The method delivered accurate predictions of removal efficiency across a range of pH values, adsorbent dosages, and contact durations, supporting the identification of optimal process conditions. The optimized approach minimized remediation costs while achieving a maximum contaminant removal efficiency of 99.49% [59]. As a category of non-biodegradable contaminants, microplastics have driven the application of machine learning in the exploitation of innovative bioremediation strategies. For instance, flagship research programs including the EU’s Horizon Europe have integrated artificial intelligence and machine learning algorithms into contaminated site management frameworks, with a focus on the sustainable regulation of emerging contaminants represented by microplastics. Through the fusion of multi-source datasets and physicochemical models, machine learning algorithms can simulate remediation processes and forecast the long-term environmental impacts of diverse treatment approaches, including biostimulation, phytoremediation, and chemical immobilization, within specific environmental matrices. In the meantime, such algorithms can adopt optimization tools to screen process parameters for high-efficiency performance, to develop more flexible and operable remediation schemes. These schemes mitigate ecological hazards while guaranteeing favorable environmental outcomes [60].

A comparable optimization strategy has also been adopted for the design of peptide-derived biomaterials that form robust binding interactions with diverse plastic substrates. Through the integration of molecular dynamics simulations and machine learning algorithms including Monte Carlo tree search, investigators can iteratively tune and optimize peptide sequences to substantially boost their microplastic adsorption capacity, which provides novel perspectives for the exploitation of targeted remediation technologies [61]. In addition, interpretable machine learning algorithms have attracted growing attention. For instance, the Shapley additive explanations (SHAP) approach, a game theory-based tool that fairly quantifies the contribution of each input feature to prediction outputs and demystifies the black-box nature of machine learning models, can both forecast the remediation performance of biochar composites and quantify the specific impacts of critical environmental variables including soil C/N ratio and clay content on remediation efficiency. This provides a theoretical basis for developing more accurate, site-adaptive remediation strategies [62]. Nevertheless, the machine learning algorithms adopted in that research merely accomplished condition optimization and performance prediction relying on controlled laboratory datasets. These models remain insufficiently validated under complicated actual aqueous environmental conditions. Furthermore, neither multi-algorithm comparative analysis nor multi-objective optimization was implemented, which imposes restrictions on the capacity for generalization and the adaptability of the constructed models to different scenarios. Furthermore, relevant studies have proposed the application of dictionary learning for FTIR-spectrum preprocessing to eliminate interference signals derived from membrane filters and enhance the identification performance of plastic contaminants. Under low signal-to-noise ratio circumstances, this approach delivers roughly 1.5-fold superior performance relative to baseline algorithms, exhibits comparable accuracy to state-of-the-art models including UNet, and possesses superior interpretability [63].

Taken together, machine learning covers the full workflow of microplastic contamination monitoring, remediation material development, and process parameter optimization, serving as a powerful tool to address the challenges of microplastic management. Nevertheless, current models suffer from insufficient scenario adaptability and a lack of validation under actual field conditions. Future research should integrate multi-algorithm comparisons and in situ measured data to further refine the model system.

### 3.2. Machine Learning in Inorganic Contaminants

Apart from organic contaminants, soil matrices harbor heavy metals including lead (Pb), cadmium (Cd), and mercury (Hg), the metalloid arsenic (As), and inorganic contaminants such as fluorides and selenium. The majority of these contaminants stem from industrial, agricultural and waste disposal activities. They feature low degradability and gradual environmental accumulation, thereby imposing long-term hazards on soil ecosystems and human health [64,65]. Conventional physical and chemical remediation methods face significant challenges when treating soils co-contaminated with heavy metals and inorganic non-metallic pollutants. They incur high economic costs, tend to damage soil structure, and carry risks of secondary contamination. Furthermore, they fail to account for the spatial heterogeneity of contamination and the differentiated remediation needs of diverse pollutants, and suffer from limitations including low treatment efficiency, poor stability, and heavy reliance on empirical operation. For instance, the application of conventional chemical leaching to extract heavy metals from soil may trigger the leaching loss of soluble phosphorus and further aggravate eutrophication in adjacent aqueous media, which reveals the inherent drawbacks of conventional remediation approaches [66].

In recent years, machine learning has been used to study soils co-contaminated with heavy metals and inorganic non-metals, for their remediation. It delivers prominent advantages in multi-factor coupled pollution scenarios. By analyzing abundant experimental and monitoring data, these models can identify key remediation factors and their interactions and transform the development of remediation schemes from empirical judgment to data-driven decision-making. This approach is particularly suitable for treating inorganic non-metallic contaminants with complex remediation mechanisms, with core applications covering contaminated site risk zoning, optimization of remediation materials and process parameters, simulation of contaminant migration and transformation, and evaluation of the long-term stability of remediation performance, as well as potential ecological risks [67,68]. For example, in chemical fixation remediation, models based on ensemble learning can accurately predict the immobilization efficiency of different biochars for specific heavy metals and can also evaluate their adsorption efficiency for inorganic non-metallic contaminants, while quantifying key influencing factors such as heavy metal type, biochar pH value, and biochar dosage [69,70]. In the field of phytoremediation, machine learning can also help identify highly enriched plants and optimize growing conditions, thereby improving restoration efficiency [71]. In summary, machine learning provides scientific, economical and sustainable technical support for the remediation of heavy metal and inorganic non-metallic contamination. Typical relevant research cases are presented in Table 2.

**Table 2:** Typical research cases of machine learning application in soil inorganic contaminant remediation.

Contaminant(s) Under Study	Sample Size	Model(s) Used	Model Input Parameters	Model Accuracy Parameters	Objective	Result	Reference
Heavy metals	30 experimental datasets (5 soil layers × 6 time points); 25 for training, 5 for validation; 7-fold cross-validation	GP, Sobol, EK, BPNN	System current, pH and electrical conductivity of anolyte/catholyte and each soil layer, soil moisture of each layer, voltage of anolyte/catholyte and each soil layer	R^2^, SAE, a1-index	GP for Cr(VI) and total Cr prediction and model evaluation	High-accuracy GP model surpasses BPNN, reflects soil Cr spatiotemporal changes	[72]
Nickel ion	662 experimental records from 124 studies; 9:1 stratified split into training and test sets; 5-fold cross-validation	CatBoost, XGBoost	Soil properties (pH, EC, CEC, organic matter, organic carbon, CaCO_3_, sand/silt/clay fractions); solution properties (initial Ni concentration, solution pH, temperature, ionic strength, solid-liquid ratio)	R^2^, RMSE, SHAP value, and feature importance score.	Model comparison for Ni^2+^ adsorption; SHAP analysis	CatBoost is superior to XGBoost (R^2^: 0.85 vs. 0.83); lower error, better generalization.	[73]
Heavy metals	215 observations from 79 studies (134 for Cd remediation); 8:2 split into training and test sets; 5-fold cross-validation	MTDL, STNN, RF, XGB, kNN, SHAP	Biochar properties (C, H, O, N content, pH, ash content, specific surface area, CEC); soil properties (pH, CEC, soil organic carbon, C/N ratio); biochar application rate	R^2^, RMSE	Multi-task DL model analyzes Cd control factors, superior to traditional models	MTDL: R^2^ = 0.70, Cd immobilization rate 62.5%, optimized biochar performs better at 10 sites	[74]
Pb	99 samples (33 treatments × 3 replicates); augmented to 1000 datasets via Gaussian mixture model	RF, XGBoost, LGBM, GMM, SHAP, t-SNE	Soil pH, EC, total nitrogen, total carbon, exchangeable K^+^/Ca^2+^/Mg^2+^/Na^+^, available phosphorus, available Pb content	R^2^, MAE, MSE, SCC, PCA	Data augmentation + ML framework: Predict long-term Pb fixation efficiency	LGBM: Optimal performance (R^2^ = 0.99); predicts long-term Pb fixation efficiency with augmented data	[75]
Cd	463 data points from 32 published studies; 8:2 randomly split into training and test sets	DT, RF, LSBoost, XGBoost, AB, CB	Soil properties (pH, EC, CEC, organic carbon, initial total Cd, moisture); biochar properties (pyrolysis temperature, elemental ratios, pH, EC, ash content, specific surface area, Cd content); experimental conditions (application rate, equilibration time)	R^2^, RMSE, MSE, PCC, HM	Cd fixation model, Bayesian optimization, and AI-assisted experimental design	LSBoost for Cd fixation analysis, AI-GUI realizes automatic experiment design	[76]
Cd	47 batch experiments (central composite design); two validation schemes (full dataset and 7:3 split)	KNN, RSM, FC-CCD	Polyaspartic acid concentration, polyaspartic acid-soil mass ratio, initial soil Cd concentration, initial pH, washing time	R^2^, RMSE	KNN and RSM for Cd-related prediction	KNN outperforms RSM (R^2^ = 0.984–0.999, lower RMSE)	[77]
Cr	420 datasets (240 for MgAl-LDH, 180 for CaAl-LDH); 8:2 randomly split into training and test sets	KNN, RF, BPNN, KAN	Environmental/process parameters (initial Cr(VI) concentration, LDH dosage, adsorption pH, temperature, contact time); LDH structural parameters (specific surface area, bond energy ratio, bond lengths)	R^2^, RMSE	modeling and factor analysis with SHAP	Ca-Al LDH outperforms Mg-Al LDH for Cr(VI) removal, key factors analyzed via SHAP	[78]
Heavy Metals	185 experimental test datasets; 8:2 randomly split into training and test sets; 10-fold cross-validation	MLR, GBoosting, XGB, Bagging	Electrokinetic remediation parameters (remediation duration, voltage, polarity reversal interval, electrolyte type, electrode area/distance/configuration); soil properties (pH, sand/silt/clay content, moisture); experimental conditions (initial heavy metal concentration, metal type, temperature)	R^2^, RMSE, MAE	ML prediction for electrodynamic remediation	Optimized XGBoost (R^2^ = 0.82); key factors ranked, favorable conditions clarified	[79]
Heavy Metals	173 data points from 20 published studies; 8:2 split into training and test sets; 5-fold cross-validation	XGBoost, ANN, KNN, 5-FCV	Plant families (7 categories); experimental conditions (temperature difference, cultivation time, pot depth, soil mass); soil properties (pH, CEC, organic carbon); metal properties (electronegativity, ionic radius, total heavy metal concentration)	R^2^, RMSE, Accuracy Score	ML models for predicting phytoremediation key indicators	XGBoost regression models achieved high prediction accuracy (R^2^ = 0.79–0.93)	[80]
Cd	822 datasets from 88 studies (incubation, pot and field experiments)	RF, SEM	Biochar properties (modification method, feedstock, pyrolysis temperature, pH, specific surface area, pore size, elemental ratios, application rate); initial soil properties (pH, CEC, organic matter, available P/K, total N); experimental conditions (duration, experiment type)	R^2^, χ^2^, df, AIC, ln (RR)	Random forests for key Cd immobilization drivers	RF identifies key factors; optimal conditions determined for Cd immobilization	[81]
Heavy Metals	29 Box–Behnken response surface runs; augmented to 87 datasets by triplication	DT, RF, SVMR, RSM, DE	Equipment operation parameters: rotational speed, homogenisation time, homogenisation pitch, descent speed	R^2^, MSE, MAE, MAPE	DE simulations verify parameter reliability	Decision Tree determines operational parameters: 73 rpm, 183 mm, 1 s, 0.06 m/s	[82]
Cr	250 experimental data points; 60 additional independent experiments for external validation	LightGBM, Adaboost, RF, XGBoost, GBDT, ANN, DT, SVR, LR	Soil physicochemical properties (pH, moisture, bulk density, organic matter, CEC, available P, exchangeable K, reactive Fe/Al/Mn oxides, particle size fractions); Cr fractionation (7 binding forms); washing agent type	R^2^, RMSE, MAE, C/C0	ML predicts Cr after leaching, field-verified	AdaBoost superior to Light GBM & RF; optimized model (R^2^ = 0.801) based on 60 samples	[83]
NaCN, HCN	82 waste samples	ANN, MLP, SLR	Initial cyanide concentration, pH, temperature, humidity, chloride concentration, alkalinity, electrical conductivity, precipitation, evaporation, time	R^2^, MSE, r	ANN for cyanide degradation and pH prediction	MLP achieves 86% accuracy for cyanide degradation prediction	[84]
Heavy Metals	29 Box–Behnken response surface runs; augmented to 290 datasets by 10 replications; supplemented with field prototype test in Liaoning	DT, RF, SVR, KNN, XGBoost, RSM, DE	Equipment structural and operation parameters: homogenisation time, homogenisation pitch, crusher spacing, rotational speed	R^2^, MAPE, MAE, MSE	On-site tests validate optimal parameters for equipment design and application	Decision Tree optimizes parameters; prototype test shows good consistency	[85]
Heavy Metals	211 pot and field experimental datasets; 7.5:2.5 split into training and test sets; 5-fold cross-validation	LR, PLS, SVR, RF, BAF	Soil properties (particle size fractions, pH, organic carbon, total/available heavy metals, metal species); biochar properties (application rate, feedstock, pyrolysis temperature); crop characteristics (experiment scale, crop type, growth period)	R^2^, MSE, RMSE, MAE	Model comparison for biochar performance prediction	RF performs best (R^2^ = 0.5924–0.7338); soil properties contribute 79.7%	[86]
Heavy Metals	322 observational datasets from 46 published studies	RF, LR, SHAP	Sulfur management (type, dosage, water regime); soil properties (pH, soil organic matter, total Fe, total S, total Cd, total As)	R^2^, RMSE, RR, CI	ML predicts sulfur effects on Cd dynamics and rice yield	RF outperforms linear regression in multiple indicators	[87]

(Note: GP = Gaussian process; Sobol = Sobol sensitivity analysis; EK = ensemble kriging; BPNN = back-propagation neural network; SAE = stacked autoencoder; CatBoost = categorical boosting; MTDL = multi-task deep learning; STNN = spatio-temporal neural network; KNN = K-nearest neighbors; LGBM = light gradient boosting machine; GMM = Gaussian mixture model; t-SNE = t-distributed stochastic neighbor embedding; MAE = mean absolute error; SCC = Spearman correlation coefficient; PCA = principal component analysis; LSBoost = least squares boosting; AB = adaptive boosting; MSE = mean squared error; HM = hyperparameter model; RSM = response surface methodology; FC-CCD = face-centered central composite design; KAN = Kolmogorov–Arnold network; MLR = multiple linear regression; GBoosting = gradient boosting; Bagging = bootstrap aggregating; 5-FCV = 5-fold cross validation; SEM = standard error of the mean; SVMR = support vector machine regression; DE = differential evolution; MAPE = mean absolute percentage error; GBDT = gradient-boosting decision tree; MLP = multi-layer perceptron; SLR = simple linear regression; PLS = partial least squares; BAF = boosting aggregation framework; RR = ridge regression; CI = confidence interval; X2 = Chi-square; df = degrees of freedom; AIC = Akaike Information Criterion; ln(RR) = logarithm of ridge regression).

Synthesis of the studies listed in Table 2 shows clear cross-cutting trends. Tree-based ensemble models including CatBoost, XGBoost, random forest, and LightGBM generally achieve superior performance for predicting heavy metal immobilization and removal efficiency, with R^2^ values ranging from 0.80 to 0.99 in most cases. Cadmium, lead, and chromium are the most extensively studied contaminants, while research on nickel, arsenic, and nonmetallic inorganic pollutants such as fluoride and selenium remains less developed. Biochar assisted immobilization, electrokinetic remediation and phytoremediation represent the three dominant application scenarios. Current models mostly focus on static endpoint prediction of remediation efficiency, and dynamic modeling of long-term contaminant transformation processes remains an underexplored direction.

#### 3.2.1. Identification, Risk Assessment and Remediation of Heavy Metal and Metalloid Contaminants

Within heavy metal remediation research, a thorough understanding of contaminant spatial distribution, source fingerprints, and their transport and transformation processes in soil matrices is essential for risk assessment and remediation strategy development. Conventional analytical methods typically face limitations of low efficiency and high costs in this field, and struggle to capture the complex nonlinear relationships underlying contamination processes. In recent years, supported by advances in machine learning, algorithms including support vector machines (SVMs), random forest (RF), and decision trees (DTs) have become key tools for optimizing heavy metal contamination management, thanks to their ability to process large datasets and identify latent distribution patterns.

In studies on contaminated site remediation, machine learning algorithms have seen increasing application to improve the efficiency of contaminant discrimination and source apportionment. For instance, the combination of spectroscopic measurements and machine learning algorithms such as random forest supports the development of large-scale classification models. These models can rapidly delineate regions with latent contamination risks and precisely identify contamination hotspots. Qi et al. [88] employed random forest to establish the first continent-wide classification model targeting four heavy metals, namely, chromium, copper, lead, and nickel, on the basis of an extensive soil spectral dataset. The area under the receiver operating characteristic curve (AUC) of the constructed model varied from 0.84 to 0.90, demonstrating its robust capacity to discriminate zones with latent contamination risks across the United States and Europe. Nevertheless, existing large-scale investigations into soil heavy metal discrimination via spectroscopic approaches are universally subject to multiple inherent constraints. Such investigations attach inadequate importance to the interactive effects between soil physicochemical attributes and heavy metals. Additionally, they are limited by data imbalance arising from the scarcity of high-concentration specimens and poor cross-adaptability across diverse spectral libraries. Leveraging such analytical approaches, environmental regulators can efficiently pinpoint contamination hotspots and rationally allocate scarce resources to conduct monitoring and remediation operations in high-risk zones. The identification of contamination sources serves as a prerequisite for source mitigation. By integrating multivariate statistical analysis with receptor models, machine learning algorithms have elevated the precision of contamination source apportionment and facilitated the execution of quantitative quantification. Li et al. [64] deployed a hybrid machine learning algorithm integrating self-organizing map (SOM) and K-means clustering. Combined with positive matrix factorization (PMF) and Monte Carlo simulation, this framework enabled qualitative source discrimination, quantitative source apportionment, and probabilistic risk evaluation for heavy metals within urban park soils. Four categories of contamination sources were distinguished in this work, verifying that mining operations and traffic discharges constitute the dominant contributors to ecological hazards. Such an integrated analytical framework mitigates the intrinsic uncertainties embedded within conventional evaluation protocols and delivers an effective technical reference for contamination source tracing, hazard management, and formulation of remediation schemes.

Machine learning algorithms can integrate datasets of soil physicochemical properties and heavy metal speciation to predict the migration behaviors of heavy metals in environmental matrices. One existing study employed random forest and support vector machine models to interpret heavy metal release behaviors in soil columns under acid rain leaching scenarios. The outputs revealed that pH and redox potential served as the dominant factors governing cadmium, lead, and zinc migration, which collectively explained more than 80% of the total variance [67]. Wang et al. [65] integrated diffusive gradients in thin-films technology with machine learning to explore discrepancies in cadmium and copper accumulation within soil–rice systems, and deployed a random forest model for precise prediction of cadmium accumulation in rice roots. Overall, machine learning algorithms can integrate contamination screening, source apportionment, and migration forecasting to support the development of comprehensive integrated risk assessment models. A research group from Lanzhou City University illustrated the integration of contamination source apportionment outputs and probabilistic risk assessment models (represented by Monte Carlo simulation). This framework enables full-scale quantitative evaluation covering the complete chain of contamination sources, transport pathways, receptor zones, and ecological hazards [64]. Furthermore, Zhang et al. [68] established an analytical approach coupling UV-Vis overlapping spectroscopy with a deep learning-based Transformer model. This strategy enables simultaneous screening of multiple heavy metals in aqueous samples and automatic conversion of concentration datasets into quantifiable ecological hazard indicators, thereby establishing an automated closed-loop workflow spanning from contamination detection to hazard assessment. Building on these technical advances, machine learning is driving the development of an intelligent closed-loop process for soil heavy metal contamination management, as shown in Figure 4.

Bioremediation achieves the uptake, transformation, and immobilization of heavy metal contaminants through plants and microorganisms, and it is widely recognized as an eco-friendly and sustainable strategy for soil remediation. Relevant studies have selected geraniums as phytoremediation plants and combined microbial application with citric acid addition to boost lead extraction efficiency. One representative study carried out experiments and model analyses across varying contamination gradients and conducted a comparative evaluation of response surface methodology (RSM) and a genetic algorithm-optimized artificial neural network (ANN-GA). Results showed that at a lead concentration of 500 mg·kg^−1^ and a 60-day cultivation period, the lead removal efficiency reached approximately 86%. By comparison, the ANN model delivered competitive predictive performance, showing promising application prospects in phytoremediation research [89]. At the stage of remediation scheme design and optimization, machine learning algorithms integrate plant physiological traits, soil physicochemical attributes, and historical remediation datasets to construct predictive models, which further screen plant species with high accumulation capacity for target heavy metals. For instance, multiple investigations adopted random forest models built on published literature datasets to identify the dominant factors governing metalloid arsenic uptake by Pteris vittata, alongside a comparative evaluation of the efficacy of various soil amendments. The outputs demonstrated that the established model achieved favorable fitting performance (R^2^ = 0.846). Plant biomass, soil amendment category, application period, and soil available arsenic content were recognized as dominant driving factors. Phosphate fertilizers exhibit superior cost-effectiveness, which can provide valuable guidance for the screening of field-scale remediation schemes [71]. Furthermore, multiple studies have selected copper as the target contaminant to compare the remediation efficacy of pure green algae cultivation and a synergistic co-culture system including Bacillus subtilis. At a copper concentration of 80 ppm, the copper removal efficiency of the single microalgae culture group reached 74.6%. For the co-cultured consortium composed of microalgae and Bacillus subtilis, the copper-removal performance increased to 84.8%. Experimental findings showed that copper inhibited algal proliferation and induced oxidative stress, while the co-culture enhanced biomass accumulation and heavy metal removal capacity. Within the concentration range of 40–80 ppm, the integrated system achieved superior remediation performance, and the established artificial neural network (ANN) model exhibited reliable predictive capacity. Collectively, such a synergistic consortium offers promising prospects for the remediation of heavy metal contaminants in aqueous media [90]. In the domains of prediction and dynamic regulation, machine learning algorithms enable real-time dynamic control of remediation processes through the fusion of in situ sensor monitoring data and advanced computational algorithms. As a representative practical case, intelligent sensing devices integrated with deep learning-based models (Bio-DANN) enable real-time monitoring of soil heavy metal levels and core environmental parameters. Corresponding regulation strategies are dynamically optimized based on model predictive outputs. This integrated closed-loop framework covering monitoring, prediction, and control ensures stable and high-efficiency remediation performance [60].

Chemical remediation, which relies on chemical amendments to immobilize and stabilize heavy metal contaminants, represents a widely adopted and efficient technique for contaminated soil treatment. Nevertheless, the screening and application dosage of remediation amendments (including biochar, alkaline solid waste, and layered double hydroxides), as well as their remediation efficacy under complex soil matrices, were conventionally determined via massive, time-intensive, and labor-costly batch experiments. Machine learning algorithms substantially reshape relevant experimental workflows through their robust data mining and modeling capabilities. During the early stages of remediation, they can develop reliable predictive models based on the intrinsic properties of soil amendments, soil physicochemical indicators, and the speciation and concentration of contaminants. Such capacities support the intelligent screening of optimal remediation agents for targeted heavy metal contamination. Likewise, for the preliminary remediation stage, existing studies have developed ten machine learning models to forecast heavy metal immobilization efficiencies. Among all the developed models, ensemble learning algorithms represented by adaptive boosting (AB) deliver the optimal fitting performance, with the maximum coefficient of determination (R^2^ = 0.96) and the minimum mean absolute error (MAE = 4.08%) and root mean square error (RMSE = 7.31%). This R^2^ value is 0.09 to 0.20 higher than results obtained from conventional linear regression models. Accordingly, such models are capable of precisely forecasting the heavy metal immobilization efficiency of alkaline solid waste and pinpointing dominant influencing factors including curing duration and CaO content, which offers straightforward guidance for the optimization of amendment mixing ratios [91]. Beyond remediation material screening, machine learning algorithms have also been demonstrated to exhibit outstanding performance in optimizing remediation process parameters. By employing regression analysis and ensemble learning approaches such as XGBoost and gradient boosting, the developed models can quantify the complex nonlinear correlations among multiple variables including reagent dosage, ambient temperature, and reaction duration. On this basis, optimal process parameters balance outstanding remediation efficacy and economic efficiency, with a maximum Cr(VI) immobilization rate up to 98.64% as a representative result. Such optimization work is not confined to individual chemical remediation processes but can also be extended to the formulation of integrated remediation schemes. For instance, relevant investigations integrated machine learning with causal inference, adopting XGBoost and SHAP to screen core factors driving cadmium accumulation in paddy soils. In this study, experimental outputs reveal that iron amendments cut root cadmium accumulation by 38.6% and efficiently lower grain cadmium concentrations by regulating available iron and pH thresholds, delivering an accurate and efficient strategy for field remediation [92]. While a decision-making framework for cadmium contamination remediation in paddy fields has been developed using machine learning and counterfactual reasoning, the framework is limited by insufficient spatiotemporal sampling coverage, failure to account for both crop-wide cadmium translocation and diverse environmental variables and inherent model uncertainties. Furthermore, the absence of field validation for the proposed remediation schemes results in limited generalizability and practical operability. In a study concerning biochar-based remediation of heavy metal-contaminated soil, a random forest model trained on 211 groups of experimental data indicated that soil properties dominated heavy metal immobilization efficiency with a contribution of roughly 79.7%, followed by biochar characteristics (17.1%), whereas crop traits exerted only marginal effects (3.2%). The model achieved R^2^ values of 0.73 and 0.70 for predicting bioaccumulation factors and crop heavy metal uptake separately, offering practical references for remediation process optimization [86]. Furthermore, trained machine learning models can predict and compare the pollutant removal efficacy of diverse remediation materials (including lichen-based activated carbon and chitosan flocculants) under systems where multiple heavy metals coexist. Such predictive tools assist decision-makers in selecting the optimal remediation strategy based on actual contamination conditions and treatment targets including wastewater purification and soil washing [93]. Machine learning algorithms enable intelligent screening of remediation materials, precise adjustment of process parameters, and economic evaluation of remediation schemes. Such technical strengths elevate the efficiency, predictability and economic performance of chemical remediation, and propel soil heavy metal remediation toward data-driven intelligent decision-making.

In summary, machine learning leverages multi-source datasets spanning soil physicochemical properties, spectral measurements, and experimental records to enable precise source apportionment, migration prediction, and full-chain ecological risk quantification of heavy metal contaminants. These algorithmic frameworks are applicable across both bioremediation and chemical remediation scenarios, supporting optimal amendment selection, process parameter tuning, and dynamic regulation of remediation operations. They address key limitations of conventional approaches, which struggle to resolve multi-factor coupling interactions or adapt to complex contaminated matrices, and establish an integrated intelligent system for the assessment and remediation of heavy metal-contaminated soils.

#### 3.2.2. Machine Learning for Soil Remediation of Inorganic Non-Metallic Contaminants

In addition to heavy metals, soil may also contain emerging non-metallic contaminants, such as excessive levels of fluoride and selenium. Soil contamination derived from non-metallic contaminants, mainly represented by fluoride and selenium pollution, brings severe environmental risks owing to the persistent properties, biological toxicity, and complex migration and transformation behaviors of these pollutants [60].

Taking fluoride as a typical case, its overaccumulation in soil mainly originates from industrial discharge, phosphate fertilizer application, and geogenic leaching in high-fluoride areas. Such excess accumulation directly triggers soil acidification, disruption of microbial communities, and nutrient imbalance. Selenium is an indispensable trace element for human health. Nevertheless, excess selenium in soil, typically derived from industrial activities and selenium-rich parent materials, is transformed into highly toxic selenate and selenite species that exert pronounced biotoxicity on flora and fauna and bioaccumulate along the food chain. Fluoride, selenium, and similar contaminants exist in multiple chemical forms and undergo complex migration and transformation processes. These processes are largely controlled by environmental variables including soil pH, redox potential, organic matter content, and coexisting ions, which makes traditional remediation technologies difficult to address their contamination effectively. For fluoride contamination, conventional chemical immobilization approaches such as lime and calcium-based amendments can reduce fluoride activity in the short term. Nevertheless, remediation efficacy is highly sensitive to soil pH; minor pH fluctuations may trigger re-release of immobilized fluoride, failing to sustain long-term stabilizing effects. For selenium contamination, the core of remediation consists in regulating selenium valence transformation. A typical strategy is to reduce highly toxic and mobile selenate into low-toxicity elemental selenium that is readily immobilized. Multiple environmental variables such as microbial activity, electron donor types, and temperature collectively modulate biological and chemical reduction processes. Conventional remediation strategies fail to realize precise regulation of reaction processes and product formation, accompanied by low treatment efficiency and excessive economic costs [94].

Machine learning algorithms, especially, deep learning and ensemble learning approaches, deliver robust data-driven tools to tackle the aforementioned complex nonlinear issues. Such techniques exhibit more prominent performance in the simulation and optimization of remediation processes. Trained on historical experimental datasets, models including support vector regression (SVR) and gradient boosting can capture and quantify the complex correlations among soil properties, remediation material dosage, environmental parameters (such as pH and temperature), selenium speciation efficiency, and fluoride immobilization efficiency [95]. For instance, one study employed ensemble algorithms such as gradient boosting and XGBoost, coupled with cross-validation and hyperparameter optimization, to develop models for evaluating the pollutant degradation efficacy of green-synthesized photocatalysts. The optimal model yielded an R^2^ value exceeding 0.99 and exhibited excellent predictive accuracy. Overall, these approaches effectively elucidate the correlations between reaction parameters and treatment performance while minimizing redundant experimental trials. Although widely adopted in aquatic remediation, these algorithms are also readily translatable to soil remediation, facilitating the intelligent development and process tuning of advanced functional materials for selenium catalytic transformation and fluoride immobilization via adsorption [95]. Furthermore, integrating predictive models with real-time sensor networks enables the development of dynamic remediation-decision-support systems. Using machine learning algorithms and real-time monitoring data of contaminant concentrations and soil property variations, the system can dynamically adjust remediation strategies. As demonstrated in a representative case, the system intelligently modulates nutrient supply to sustain optimal microbial activity during selenium bioremediation; for fluoride immobilization, it pre-identifies critical pH thresholds and automatically adjusts stabilizer dosing, ultimately enabling precise and adaptive control over the entire remediation process. Within such a system, the integration of machine learning algorithms and IoT sensor networks incorporating distributed sensor arrays for automatic environmental data acquisition and real-time wireless data transmission strengthens the performance of data collection, predictive analysis, and real-time response [96]. Overall, such integration not only facilitates real-time decision-making but also allows forecasting of subsequent evolution trends according to updated parameters amid varying environmental conditions.

Overall, in the remediation of non-metallic contaminants, the process is difficult to predict, the parameters are hard to optimize, and the results are hard to maintain. Owing to their capacity to identify implicit data patterns, model complex variable correlations, and predict spatiotemporal variations, machine learning algorithms are driving a shift in this field from experience-dependent regulation to data-oriented precise management.

## 4. Conclusions and Prospects

### 4.1. Advantages of Machine Learning in Soil Remediation

Soil contamination issues have grown progressively complex. Accordingly, scholars must tackle numerous variables and complex interactive correlations during the characterization of contaminant behaviors and the assessment of remediation efficacy. Compared with conventional models built upon empirical formulas and linear hypotheses, machine learning exhibits superior flexibility. Algorithms including supervised learning, unsupervised learning and deep learning can realize contaminant category discrimination, contamination degree forecasting and remediation performance evaluation, thereby offering data-driven decision support for soil contamination remediation. The overall framework of machine learning implementation in soil remediation is illustrated in Figure 5.

Contaminant migration and transformation in soil media are governed by multiple factors, including intrinsic contaminant properties, soil physicochemical characteristics, and regional climatic conditions. Correlations among these factors typically present pronounced nonlinear features, which restricts the applicability of conventional statistical methods. Algorithms including random forest (RF), support vector machines (SVMs), and neural networks (NNs) support the development of predictive models without rigid prior assumptions, thereby enhancing the precision of contamination forecasting and the robustness of remediation efficacy evaluation. For instance, in studies predicting heavy metal spatial distribution in deep soil, a random forest model yielded R^2^ values ranging from 0.757 to 0.897; these values were 0.08 to 0.22 higher than results obtained from conventional linear regression models. Meanwhile, this approach offers technical guidelines for zoning remediation, source identification of contaminants, and relevant studies on comparable contaminated sites [97]. With advances in remote sensing, sensor networks, and spectroscopic technologies, the acquisition of environmental monitoring datasets has improved significantly in terms of both efficiency and accessibility. These technological advances have facilitated the widespread adoption of machine learning for contamination discrimination and environmental surveillance. By fusing multi-source datasets, models can characterize the spatial distribution patterns of contaminants and assist in the delineation of high-risk contaminated zones. As an effective non-contact monitoring tool for regional environmental monitoring, remote sensing facilitates mass collection of multi-dimensional surface data. Even so, remote sensing (RS), a sensor-based non-contact approach for surface data collection and analysis, tends to generate datasets affected by noise disturbances. To tackle such noise interference, noise-resistant ensemble algorithms, prominently, random forest models, see extensive utilization in remote sensing data processing for environmental monitoring studies [98]. Machine learning techniques also exhibit notable advantages in the optimization of remediation processes. By analyzing historical experimental datasets and field monitoring records, models can predict the remediation performance of various amendment materials and varied dosages under specific environmental conditions, providing a theoretical and practical basis for process parameter regulation. In addition, machine learning techniques can also furnish reliable support for governance decision-making with regard to contaminated sites. By integrating contaminant source information, environmental characteristic parameters, and efficiency indicators of various remediation technologies, this approach supports comprehensive evaluation of multiple remediation alternatives and provides data-driven support for scheme decision-making. Overall, artificial intelligence has emerged as a vital tool for improving remediation efficiency [99]. This review synthesizes the recent literature to show that machine learning delivers powerful tools for addressing complex challenges within contaminated soil remediation. These approaches support contaminant characterization and remediation design, and enable site-specific management strategies, opening new avenues toward intelligent, data-centered control of polluted soils.

### 4.2. Limitations of Machine Learning in the Application of Soil Remediation

Despite the remarkable potential demonstrated by machine learning in soil contamination remediation, numerous challenges remain to be addressed for practical on-site implementation. These challenges can be grouped into three categories: data-related constraints, pollutant-soil matrix complexities, and practical obstacles across remediation stages. First and foremost, raw datasets present inherent limitations. Experimental datasets are generally generated by independent research groups, with substantial discrepancies in experimental setups and operational parameters, as well as a lack of unified standardized protocols. Such inconsistency hinders the direct fusion of multi-source datasets and restricts the conduct of valid comparative analyses. Apart from the substantial costs associated with environmental monitoring and laboratory analytical tests, the scarcity of training samples also negatively impacts model generalization capacity and the reliability of on-site predictive outputs. Published research indicates that challenging data collection, a lack of unified standardization, and inadequate data sharing channels represent key constraints for machine learning deployment in environmental research [98]. The interpretability of machine learning models remains a critical unresolved challenge. Advanced algorithms represented by deep neural networks and ensemble learning achieve strong predictive accuracy. Nevertheless, their internal decision-making logic lacks sufficient interpretability, and such algorithms are therefore widely referred to as “black-box models”. In practical soil contamination remediation projects, overreliance on model predictions without accounting for contaminant transport mechanisms and key controlling factors may undermine the scientific rationality and reliability of formulated remediation schemes. In response to poor model interpretability, growing research efforts have focused on interpretable machine learning techniques to break down the “black box” and furnish clear explanatory references for environmental decision formulation. For instance, SHAP (Shapley additive explanations) value analysis quantifies the marginal contribution of each input feature to model predictive outputs, which allows explicit identification of dominant factors governing pollutant removal efficiency and overall remediation performance, including soil pH, soil organic matter content, and amendment dosage. In turn, the LIME (local interpretable model-agnostic explanations) framework produces local explanatory interpretations for individual samples, uncovering the specific internal logic supporting model predictions under site-specific contamination scenarios. Collectively, these interpretability approaches can not only enhance the reliability of model-derived conclusions but also provide interpretable quantitative evidence to support the optimization of remediation operational parameters and the formulation of site-specific remediation strategies. Furthermore, model overfitting and data leakage constitute another set of potential risks. When training datasets share strong correlations with test datasets, the predictive capacity of such models tends to be overestimated, which hinders the reproducibility of favorable outcomes on unseen datasets and practical field conditions. Accordingly, establishing a rational data partitioning strategy and adopting cross-validation to evaluate model stability represent core approaches to guarantee the reliability of predictive results [100].

Beyond data-related constraints, challenges also arise from pollutant and soil matrix conditions, as well as the practical requirements of real-world remediation workflows. Soil physicochemical properties, climatic conditions, and pollution sources differ drastically across geographic regions, which means that models trained on region-specific datasets cannot be directly extended to other areas. From the perspective of field application, gaps still exist between laboratory-derived model outputs and fluctuating on-site conditions across multiple remediation phases. Accordingly, enhancing the generalization capacity of these models and enabling their adaptation to heterogeneous environmental conditions represents a key direction for subsequent research.

This narrative review also has several inherent limitations. At present, there is no universally recognized standardized quality-assessment framework specifically for environmental machine learning research. The publications included in this review exhibit high heterogeneity in sample size, dataset sources, experimental design, and model validation strategies. In the absence of domain specific evaluation tools, quantitative grading or scoring of all retrieved studies cannot be implemented in this work. Original conceptual models specially tailored for machine learning-driven soil remediation have not been developed within this review, and constructing such conceptual frameworks represents a promising direction for future investigations.

### 4.3. Prospects

For future research aimed at advancing the practical application of machine learning in soil remediation, systematic optimization of its full application ecosystem is urgently needed. Rather than pursuing overly generalized unified specifications for global data sharing, more focus should be placed on heterogeneous data fusion. This framework integrates laboratory experimental datasets, discrete field sampling records, and site monitoring information across varied soil backgrounds and pollution conditions, to narrow the gap between laboratory data and complex field conditions. Meanwhile, full account should be taken of the regional heterogeneity of environmental parameters, and a stratified dataset system should be established to ensure training data quality and underpin the stable, reliable deployment of predictive models for contaminated soil sites. On this basis, interpretable machine learning tools including SHAP and feature importance analysis can be applied to quantify and screen core factors governing contaminant migration and degradation in real-world soil remediation systems, rather than being used as a generic routine analytical workflow. To tackle the prevalent small-sample limitation in field investigation of soil remediation, data augmentation and transfer learning techniques should be tailored to soil matrix characteristics to avoid erroneous model predictions induced by sample bias. Under the context of climate change, machine learning can cut implementation costs and improve the remediation efficiency of nature-based solutions (NbSs), thereby accelerating the large-scale application of such sustainable remediation technologies. Nevertheless, dynamic process modeling of remediation, i.e., coupling data-driven modeling with physical and chemical mechanisms of pollutant migration and remediation to simulate time-dependent pollutant transformation rather than only static endpoint results, constitutes a prominent unresolved challenge for subsequent investigations. Future research may establish dedicated quality assessment criteria for machine learning driven environmental studies, to facilitate more objective comparison and evaluation of published research outputs.

## Figures and Tables

**Figure 1:**
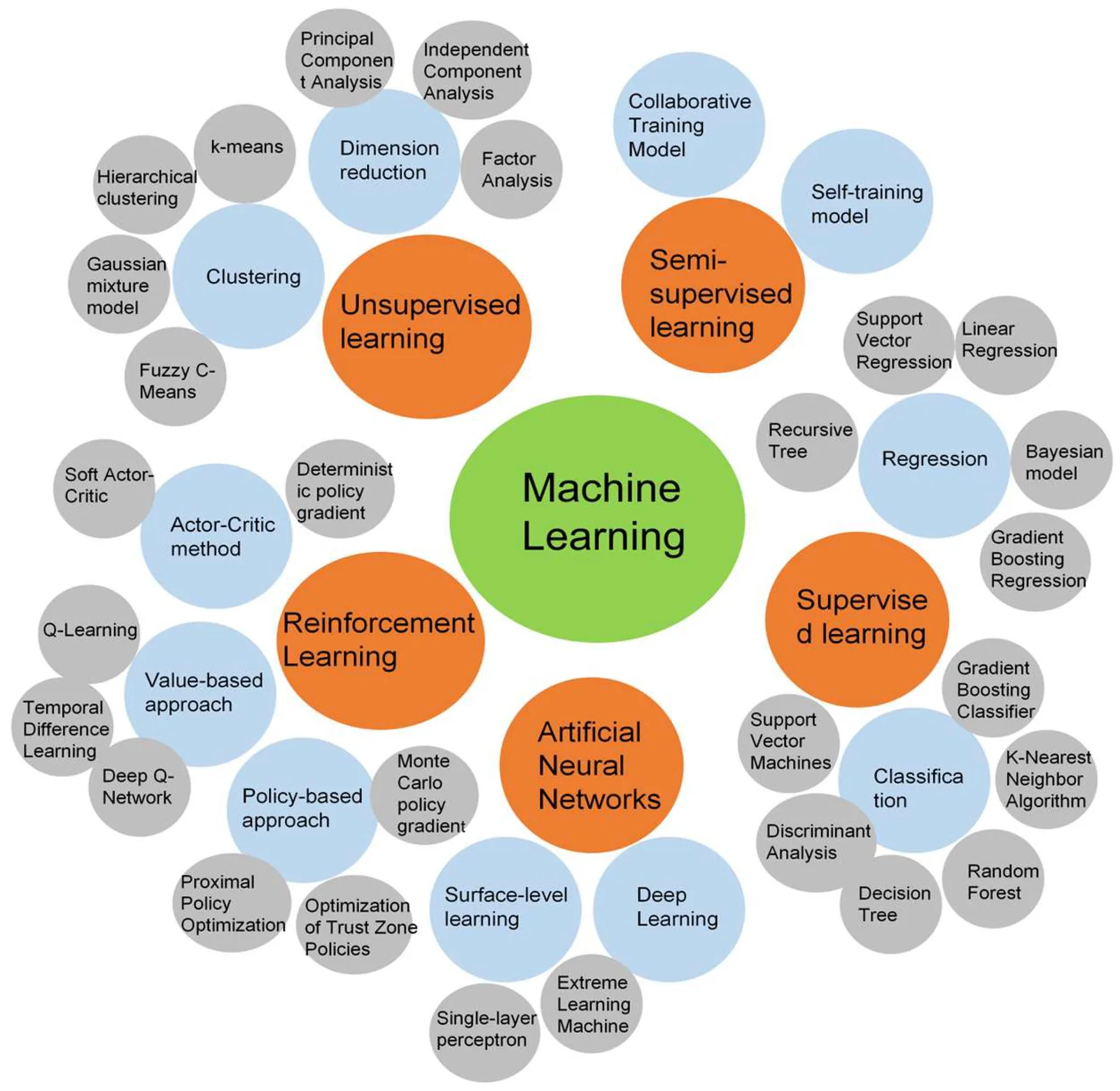
Common machine learning algorithms.

**Figure 2:**
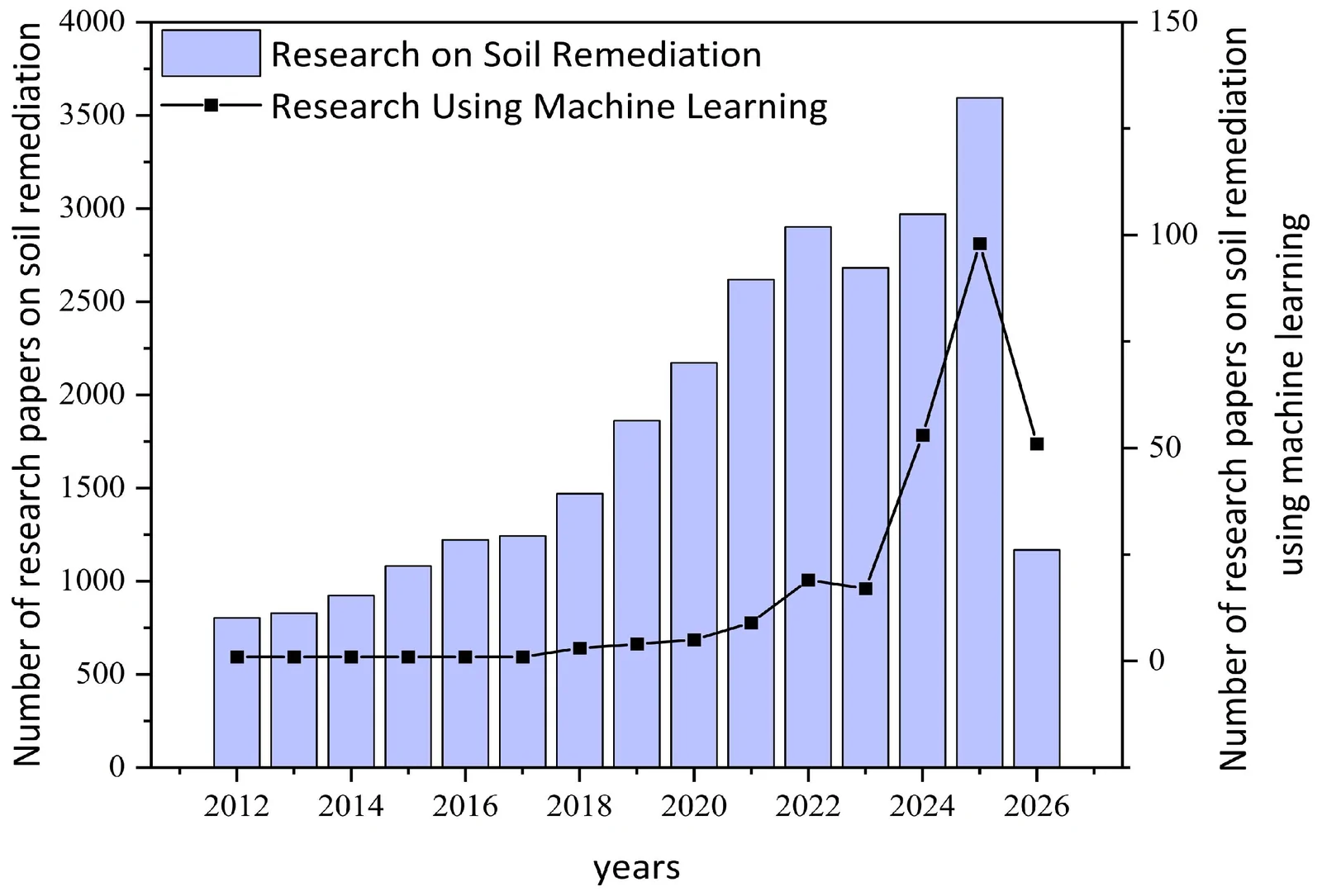
Changes in the number of publications on soil remediation research and machine learning applications (as of 30 April 2026).

**Figure 3:**
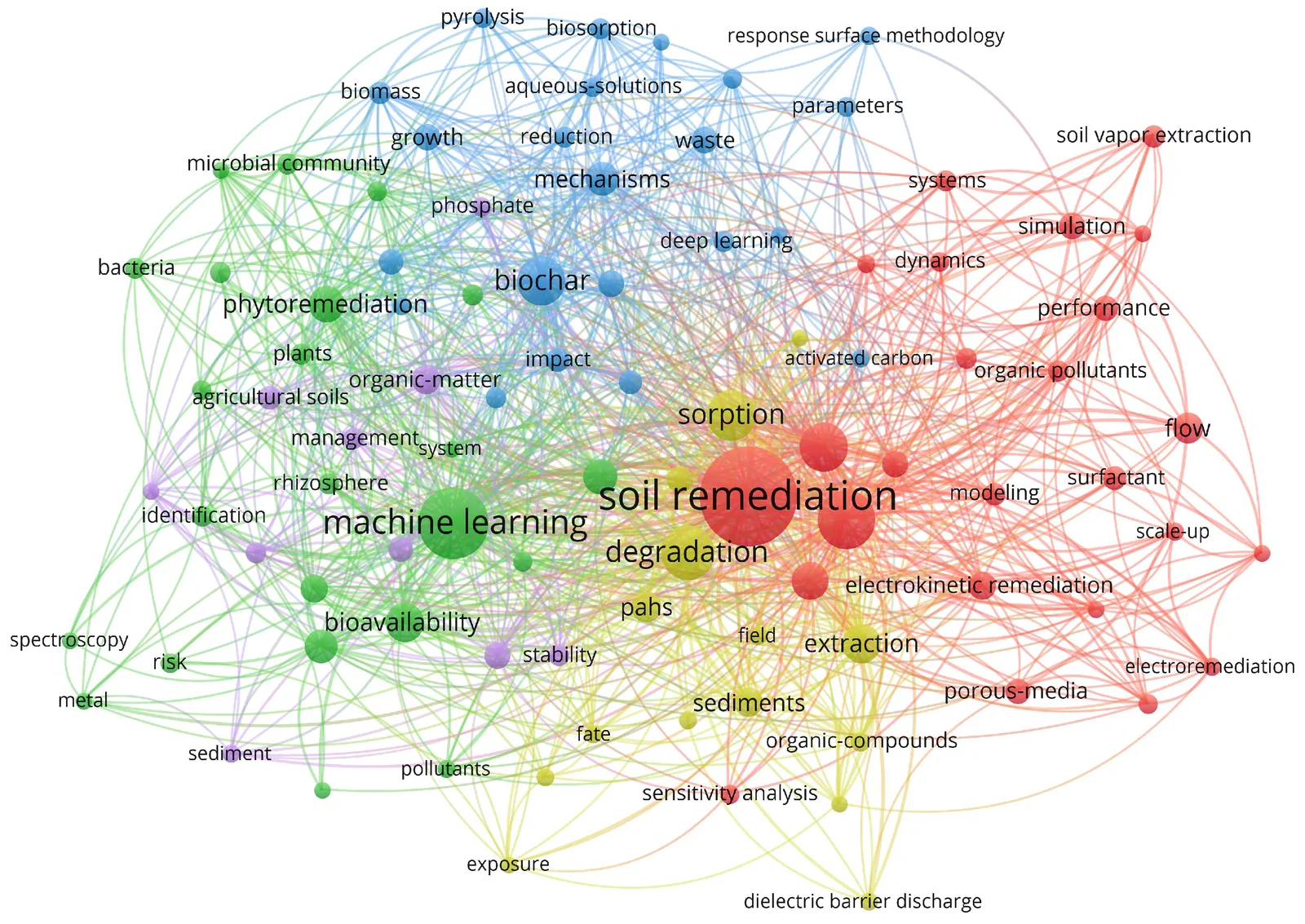
Research hotspots in machine learning for soil remediation.

**Figure 4:**
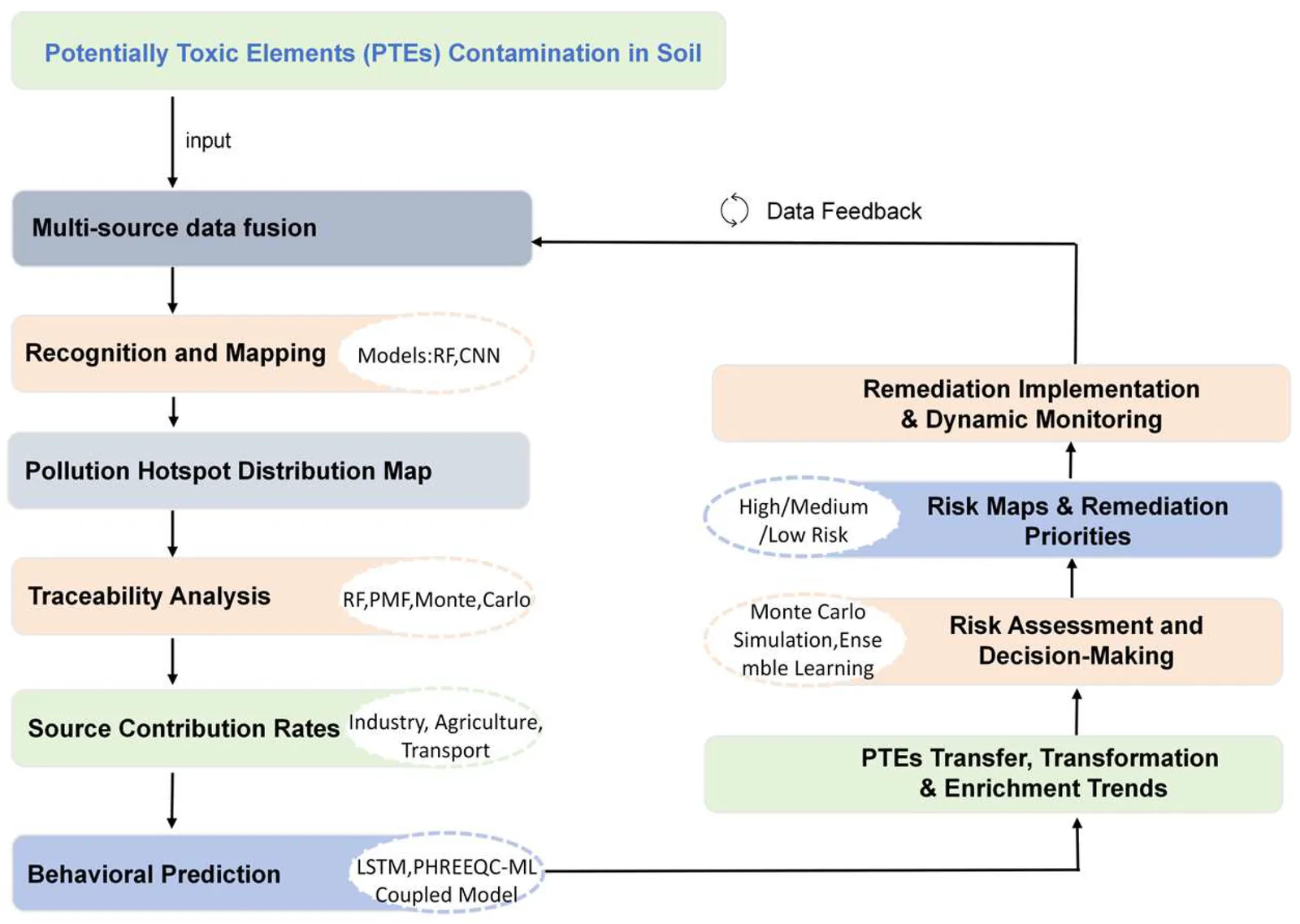
Intelligent full-chain management flowchart of soil heavy metal contamination driven by machine learning.

**Figure 5:**
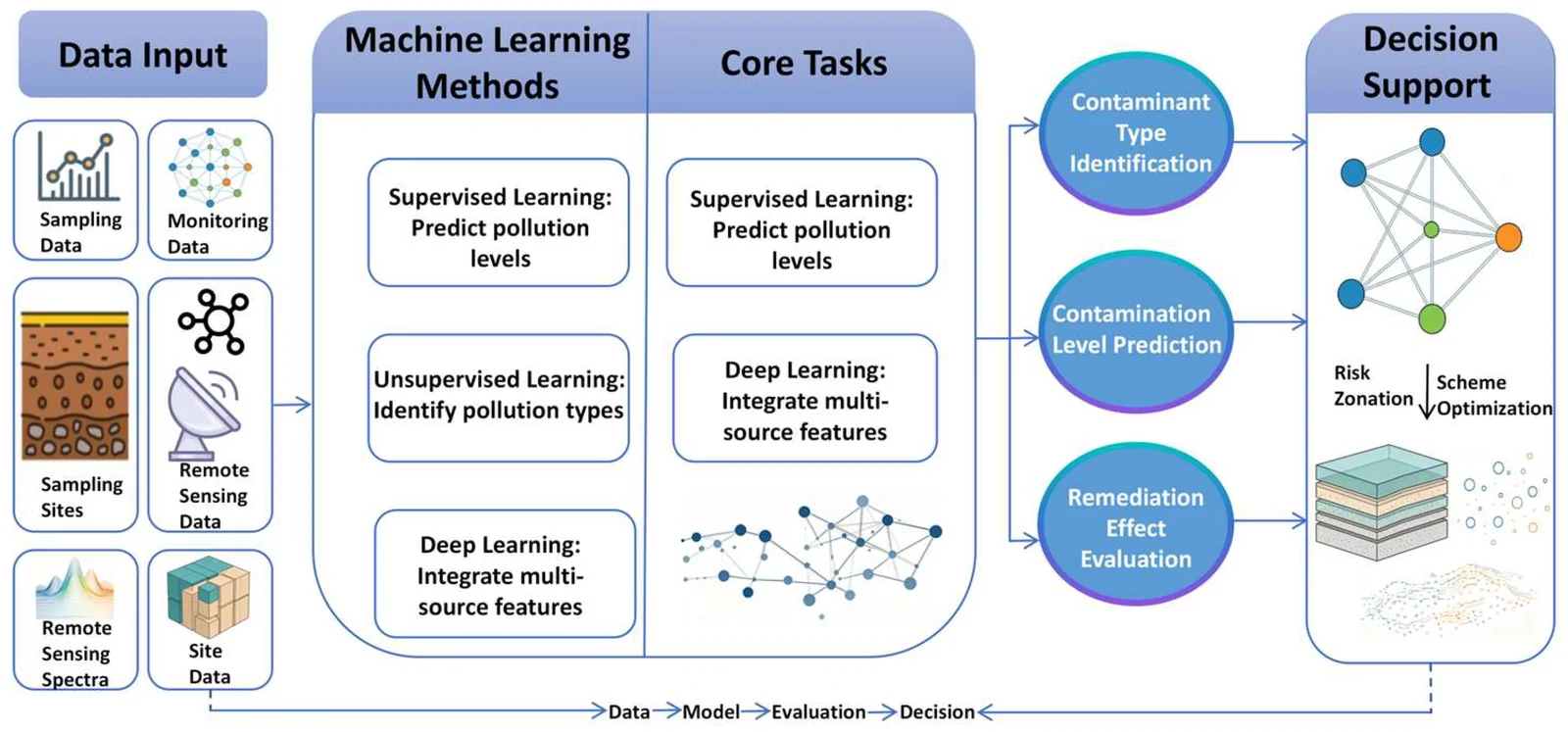
Application framework of machine learning in soil remediation.

## Data Availability

No new data were created or analyzed in this study. Data sharing is not applicable to this article.
